# IgG3 and IL10 are effective biomarkers for monitoring therapeutic effectiveness in Post Kala-Azar Dermal Leishmaniasis

**DOI:** 10.1371/journal.pntd.0009906

**Published:** 2021-11-10

**Authors:** Shilpa Sengupta, Mitali Chatterjee

**Affiliations:** Dept. of Pharmacology, Institute of Postgraduate Medical Education and Research, Kolkata, West Bengal, India; Academic Medical Center: Amsterdam UMC Locatie AMC, NETHERLANDS

## Abstract

**Background:**

The assessment of chemotherapeutic responses in Post Kala-azar Dermal Leishmaniasis (PKDL), especially its macular form is challenging, emphasizing the necessity for ‘test of cure’ tools. This study explored the diagnostic and prognostic potential of IgG subclasses and associated cytokines for monitoring the effectiveness of chemotherapy in PKDL.

**Methods:**

Participants included PKDL cases at (a) disease presentation, (b) immediately at the end of treatment (12 weeks for Miltefosine or 3 weeks for Liposomal Amphotericin B, LAmB and (c) at any time point 6 months later, for estimating anti-leishmanial immunoglobulin (Ig, IgG, IgM, IgG1, IgG2 and IgG3) and cytokines (IL-10, IL-6).

**Results:**

In PKDL, Ig levels were elevated, with IgG3 and IL-10 being the major contributors. Miltefosine decreased both markers substantially and this decrease was sustained for at least six months. In contrast, LAmB failed to decrease IgG3 and IL-10, as even after six months, their levels remained unchanged or even increased.

**Conclusions:**

In PKDL, IgG3 and IL-10 proved to be effective predictors of responsiveness to chemotherapy and may be considered as a non invasive alternative for longitudinal monitoring.

## Author summary

Post Kala-azar Dermal Leishmaniasis (PKDL) is a dermal condition that occurs in East Africa and South Asia, the latter in 5–10% of patients after apparent cure from Visceral Leishmaniasis (VL). Till date, conventional knowledge in South Asia was that the polymorphic form of PKDL comprising of macules, papules and nodules was the predominant disease form, constituting 85–90%. However, since 2014, implementation of active-case surveillance led to unearthing of a large number of macular, hypopigmented cases, and was reported to contribute to nearly 50% of the disease burden. In particular, the macular form poses a diagnostic dilemma as microscopically parasites are difficult to identify in their lesions, and repigmentation occurs months after parasite clearance, emphasizing the need for developing non-invasive approaches for measurement of parasite burden. Till date, no formal clinical trial for treatment of PKDL has been undertaken where the parasite load was quantified and treatment remains empirical. This is primarily due to PKDL cases being unwilling to provide a repeat skin biopsy once their lesions have declined. Therefore, in cases where treatment failure occurs, it cannot be precisely identified, and could potentially lead to these cases becoming mobile disease reservoirs, thereby adversely impacting on the ongoing VL elimination programme. This study addressed this critical lacuna, where it was established that in both clinical types of PKDL, circulating levels of IgG3 and IL-10 can be considered as effective markers for monitoring treatment outcome. At disease presentation, the raised levels of IgG subclasses and associated cytokines (IL-10 and IL-6) declined following therapy with Miltefosine, the maximum decrease being with IgG3 along with IL-10; importantly, this decrease was sustained for at least six months. In contrast, LAmB failed to decrease the levels of immunoglobulins and associated cytokines even six months after completion of treatment; in fact the antibody levels either increased or remained unchanged. Taken together, this study has established the potential of IgG3 and IL10 as a non-invasive alternative for monitoring of chemotherapeutic responses in PKDL.

## Introduction

Leishmaniasis, a vector-borne parasitic disease, caused by at least 20 species of the genus *Leishmania* accounts for a clinical pleomorphism that ranges from cutaneous/mucocutaneous lesions to a life threatening visceral involvement, as determined by an interplay of parasite characteristics, vector biology and host immune responses [1]. Possibly the most challenging disease form is Post Kala-azar Dermal Leishmaniasis (PKDL), a dermal sequel that occurs in patients with apparently cured Visceral Leishmaniasis (VL) [2]. Periodic peaks of VL in South Asia are considered as the norm [3], and a consensus is that during the inter-epidemic periods, PKDL cases fuel the transmission [4–6]. Owing to the poor treatment seeking behavior of patients with PKDL, they innocuously harbor parasites in their skin and serve as ‘mobile disease reservoirs’. This ensures parasite transmission in the community, making PKDL a major impediment to the ongoing South-East Asia Region Kala-azar Elimination Programme [7–9].

With implementation of active case surveillance in West Bengal, India from 2014, a huge number of macular PKDL cases were unearthed, and translated into the conventional ratio of polymorphic: macular change from 9:1 to 1:1 [10]. In particular, the macular type poses a diagnostic dilemma owing to the minimal presence of parasites, and its clinical features being indistinguishable from other hypopigmentary disorders like vitiligo, pitryasis versicolor or leprosy. Accordingly, quantification of parasite load is the sole objective parameter of efficacy, but is logistically difficult to implement as upon completion of treatment, patients are either lost to follow up or are reluctant to provide a repeat skin biopsy especially if the lesions have resolved. Therefore, as the parasite burden cannot be quantified, conducting clinical trials at least in South Asia (that includes India, Bangladesh and Nepal) is difficult, resulting in important gaps in our knowledge. Therefore, there is a need for developing non-invasive approaches for monitoring of treatment efficacy.

Miltefosine was empirically recommended for 12 weeks for PKDL, and is associated with a 15–20% relapse rate emphasizing the need for monitoring, preferably with molecular tools [11,12], while the treatment regimen for LAmB in PKDL was empirically set at 30 mg/kg b.w. for 3 weeks [11,12]. The chemotherapeutic efficacy of Miltefosine vs. LAmB was assessed by quantifying the parasite load in skin biopsies [13] wherein irrespective of the lesional type, patients following treatment with Miltefosine showed an absence of parasite DNA that was sustained up to at least 6 months; however, with LAmB there was parasite persistence suggesting treatment inadequacy [13,14]. In resource limited settings, quantification of parasite load using nucleic acid based detection methods is not always feasible, endorsing the need to validate alternatives e.g. serological approaches for developing a ‘test of cure’ [15]. Accordingly this study explored the prognostic potential of measuring IgG subclass responses and cytokine levels for monitoring responses.

## Material and methods

### Ethics statement

The study was approved by the Institutional Ethics committees of School of Tropical Medicine, Kolkata and IPGME&R, Kolkata, India; written informed consent was obtained from the individual or their legal representative to publish photographic images of the lesions.

### Study population

**Fig 1:**
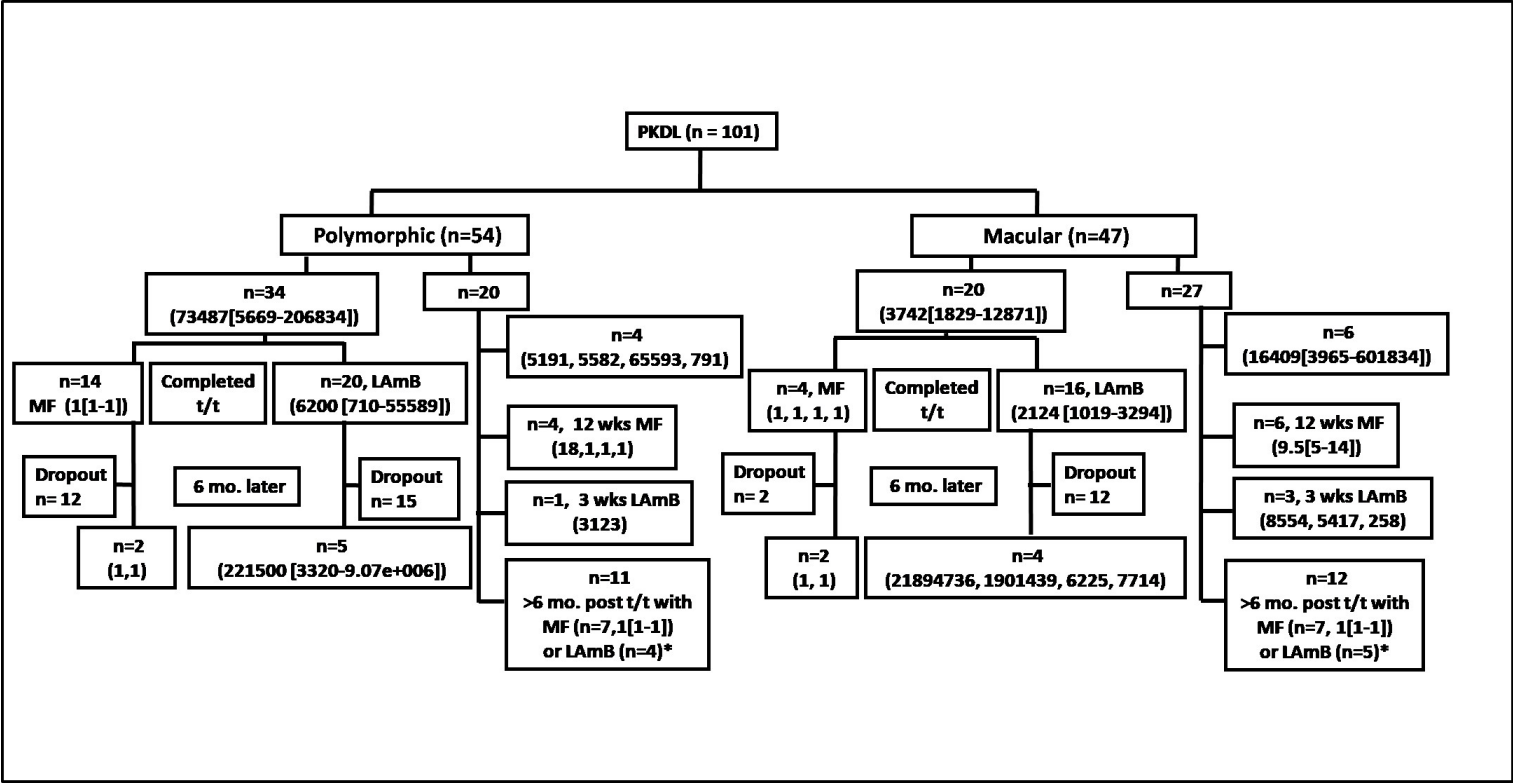
Schematic diagram indicating patient recruitment and their parasite load; drop out indicates PKDL cases not willing to provide a skin biopsy/blood at the indicated time point. *Patients did not agree to provide biopsy, only gave peripheral blood.

This study included PKDL cases (n = 101) sourced from passive surveillance (n = 24), where patients presented at the Dermatology Outpatient Department of School of Tropical Medicine, Calcutta Medical College and Institute of Post Graduate Medical Education & Research, IPGME&R, Kolkata, India (n = 24, 2009–2015), or following active surveillance (n = 77) in VL hyper-endemic districts of West Bengal, e.g. Malda, Dakshin Dinajpur, Murshidabad and Birbhum from 2015–2020 (**Fig 1**) [10]. Using standard case definitions and defined risk factors e.g. living in an endemic area and having an epidemiological link (past history of VL), they were sub grouped into polymorphic and macular PKDL, with an initial diagnosis based on clinical features, a prior history of VL, rK-39 positivity and were confirmed by ITS-1 PCR and/or Giemsa staining for the presence of Leishman Donovan bodies [16]. None of the patients had any co-infections or pre-existing disease. As controls, 15 healthy volunteers were recruited from endemic and non-endemic areas; their ages were comparable and were seronegative for anti-leishmanial antibodies. However, as the ratio of male: female varied depending on the type of surveillance, a gender-match for healthy controls was not done. After confirmation by ITS-1 PCR, patients were randomly allocated to receive Miltefosine or LAmB and samples collected at disease presentation, immediately on completion of treatment with Miltefosine (for 12 weeks) or LAmB (for 3 weeks), and at the end of six months post-treatment (**Fig 1)**. Among them, serial monitoring was achievable in 18 and 36 cases who received Miltefosine and LAmB respectively (**Fig 1)**. On a lesional basis, serial monitoring was done in polymorphic (n = 34) or macular (n = 20) cases.

### Reagents

All reagents were obtained from Sigma (St. Louis, MO, USA) except anti-human IgG3-HRP from Invitrogen, (Waltham, MA, USA), ELISA kits for IL-10 and IL6 from Immunotools (Friesoythe, Germany), Protease inhibitors and 2,2’-azino-bis[3-ethylbenzthiazoline- 6-sulfonic acid] (ABTS) from Roche Applied Sciences (Penzberg, Germany), bovine serum albumin (BSA) from Himedia (Mumbai, India) and polystyrene coated maxisorp strips (Nunc Immunomodules, Roskilde, Denmark).

### Preparation of crude *Leishmania* lysate

Crude antigen (LDA) lysate was prepared from a *L*. *donovani* strain MHOM/IN/1983/AG83 using log phase promastigotes as previously described [17]. Lysates were stored at -20°C until use, and just prior to use diluted in phosphate buffer (0.02 mol/L, pH 7.8) which served as the coating antigen.

### ELISA for total anti-leishmanial Ig, IgG and IgM

LDA was added to polystyrene coated wells (1μg/well/100 μl), incubated overnight at 4°C, and following three washings with PBS supplemented with 0.05% Tween-20 (PBS-T); the nonspecific binding sites were blocked by PBS supplemented with 2% BSA (PBS-BSA). After an overnight incubation at 4°C with plasma (1:500 in PBS-BSA; 100 μl/well), binding was detected using horse radish peroxidase (HRP) conjugated protein-A (1:6000 in PBS-BSA, 100 μl/well) or anti human HRP-IgG (1:15,000 in PBS-BSA, 100 μl/well) or HRP-IgM (1:10,000 in PBS-BSA, 100 μl/well) and incubated at 37°C for 30 min. Binding was detected using ABTS and OD_405_ nm measured on an ELISA reader (Merilyzer EIAQuant, Meril Life Sciences, India).

### Measurement of antileishmanial IgG subclasses

For determination of IgG subclasses, wells coated with LDA were incubated overnight with plasma (1:50 in PBS-BSA), followed by incubation with biotinylated anti human IgG1 (1:1000 in PBS-BSA) or biotinylated anti human IgG2 (1:15,000 in PBS-BSA) for 30 minutes at 37°C, and binding detected using streptavidin-HRP (1:15,000 in PBS-BSA). For IgG3, plasma (1:10 in PBS-BSA) was incubated overnight at 4°C with anti-human IgG3-HRP (1:500 in PBS-BSA), and binding detected using ABTS (100 μl) as the substrate and absorbances measured at 405 nm.

### Cytokine ELISA

Levels of cytokines (IL-10, IL-6) were measured using commercially available kits (Immunotools Friesoythe, Germany). Briefly, the individual cytokine antibodies were coated and after blocking of nonspecific sites with PBS-BSA, individual cytokine standards/plasma was added followed by biotinylated anti-cytokine antibodies, and binding detected using streptavidin-HRP (1:1000 in PBS-BSA) and TMB (100 μl) as the substrate, followed by stop solution (1M H_2_SO_4_) and absorbances measured at O.D.405nm. The sensitivity of detection of IL-10 and IL-6 was 9.4 and 6.1 pg/ml respectively.

### Statistical analysis

Results were expressed as median (Interquartile range, IQR), and data analyzed between groups by Kruskal Wallis test followed by Dunn’s multiple comparison test for non-parametric data using GraphPad Prism software version 5.0 (GraphPad Software Inc., La Jolla, CA, USA); p<0.05 was considered as significant.

## Results

### Study population

Presently, the distribution of polymorphic and macular PKDL is around 1:1 [10], and was reflected in the study population as at disease presentation included 101 polymorphic and macular cases, (**Table 1 and Fig 1**). Amongst the polymorphic cases, 20 were from passive surveillance and 34 following active surveillance, whereas in the macular type, majority were by active case detection (n = 43), and only 4 by passive surveillance. The disease duration was comparable between the two clinical forms, being 3[1.1–6] and 2 [1–3] years for polymorphic and macular PKDL respectively. There was a male preponderance in the passive surveillance group, and their lesions were predominantly polymorphic (**Table 1)**.

**Table 1:** Study Population.

Clinical Features	Patients with PKDL (n = 101)		Healthy controls (n = 15)
	Passive Surveillance (n = 24)	Active Surveillance (n = 77)	
Age (in years)	*29(18–40)	*20(14–30)	*27 (25–35)
Gender (male: female)	5:1	4:3	1.1:1
History of VL (%)	95	92	NA
Polymorphic: macular	20:4	34:43	NA
Interval between cure of VL and onset of PKDL (in years)	*5 (3–9)	*4 (3–8)	NA
Duration of PKDL/ patient delay (in years)	*3(2–8.5)	*2 (1–4)	NA

*Values are expressed as median (IQR); NA = Not applicable; PKDL = Post Kala-azar Dermal Leishmaniasis; VL = Visceral Leishmaniasis

Following ITS-1 PCR positivity, patients were randomly allocated to receive Miltefosine or LAmB (**Fig 1**), and as a quantifiable parameter of efficacy, the parasite load was measured. Among the 54 polymorphic cases, 34 were serially monitored, and received Miltefosine (n = 14) or LAmB (n = 20). The parasite burden at disease presentation was high and declined following treatment with Miltefosine, while LAmB failed to reduce the parasite burden **(Fig 1)**. Cases who returned at any time point six months later demonstrated sustained parasite clearance with Miltefosine (n = 2), whereas with LAmB, (n = 5) the parasite load increased (**Fig 1**). The remaining 20 polymorphic cases included naïve PKDL cases (n = 4), immediately at end of treatment with Miltefosine (n = 4) or LAmB (n = 1), and >6 months completion of treatment with Miltefosine or LAmB (n = 11, **Fig 1**). Overall, Miltefosine caused a sharp reduction in parasite load whereas with LAmB, the parasite burden persisted (**Fig 1**).

Among the 47 macular cases, 20 could be serially monitored following treatment with Miltefosine (n = 4) or LAmB (n = 16). At six months, the parasite load decreased dramatically after treatment with Miltefosine and was sustained. However, LAmB failed to decrease the parasite load and instead, increased or remained unchanged at six months (**Fig 1)**. The remaining 27 macular cases included naïve cases (n = 6), immediately at end of treatment with Miltefosine (n = 6) or LAmB (n = 3), or at any time point 6 months after completion of treatment (n = 12, **Fig 1**) and the scenario was similar wherein Miltefosine showed complete parasite clearance, whereas LAmB failed to eliminate the parasites (**Fig 1**).

### Levels of antileishmanial immunoglobulin in PKDL

**Fig 2:**
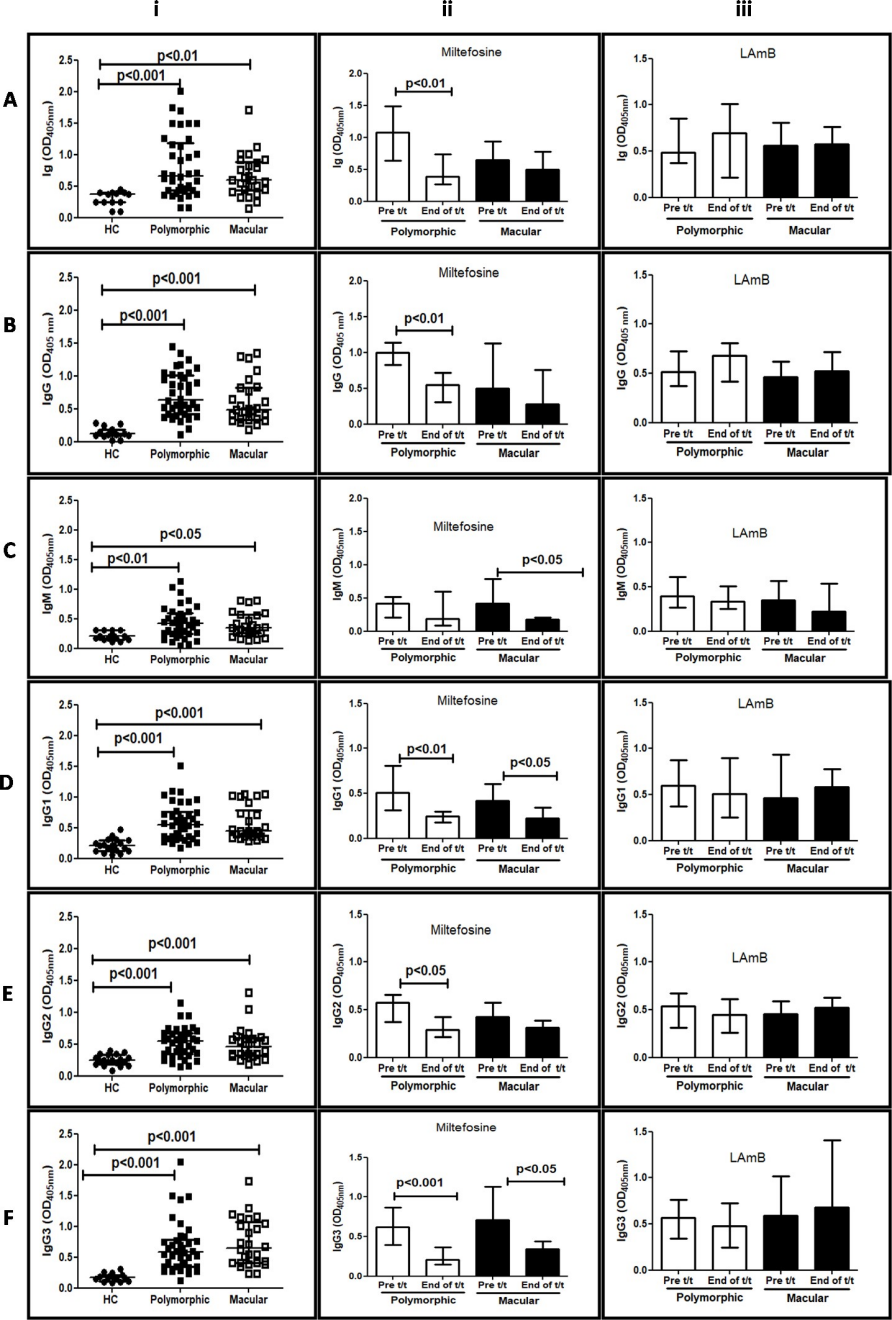
**Impact of chemotherapy upon levels of anti-leishmanial immunoglobulins in patients with PKDL A: Effect of treatment upon levels of total antileishmanial Ig in PKDL.** i Scatter plots indicating the median (IQR) of total antileishmanial Ig in patients with polymorphic PKDL (n = 38, ■), macular PKDL (n = 26, □) and healthy controls, HC (n = 15, ●) ii Bar graphs showing the median (IQR) of total anti-leishmanial Ig before (pre) and after treatment (t/t) with 12 weeks Miltefosine in patients with polymorphic (Poly, n = 18, □) and macular (Mac, n = 10, ■) PKDL. iii Bar graphs showing the median (IQR) of total anti-leishmanial Ig at disease presentation (pre) in patients with polymorphic (Poly, n = 24, □) and macular (Mac, n = 22, ■) PKDL, and after 3 weeks of treatment (t/t) with LAmB (Poly, n = 21, □) and (Mac, n = 19, ■). **B**: **Levels of total anti-leishmanial IgG following treatment in PKDL.** i Scatter plots indicating the median (IQR) of anti-leishmanial IgG in patients with polymorphic PKDL (Poly, n = 38, ■), macular PKDL (Mac, n = 26, □) and healthy controls, HC (n = 15, ●) ii Bar graphs showing the median (IQR) of anti-leishmanial IgG before (pre) and after treatment (t/t) with 12 weeks Miltefosine in patients with polymorphic (Poly, n = 18, □) and macular (Mac, n = 10, ■) PKDL. iii Bar graphs showing the median (IQR) of anti-leishmanial IgG at disease presentation (pre) in patients with polymorphic (Poly, n = 24, □) and macular (Mac, n = 22, ■) PKDL, and after 3 weeks treatment (t/t) with LAmB (Poly, n = 21, □) and (Mac, n = 19, ■). **C: Levels of total antileishmanial IgM following treatment in PKDL** i Scatter plots indicating the median (IQR) of anti-leishmanial IgM in patients with polymorphic PKDL (n = 38, ■), macular PKDL (n = 26, □) and healthy controls HC (n = 15, ●) ii Bar graphs showing the median (IQR) of anti-leishmanial IgM before and after treatment with 12 weeks Miltefosine in patients with polymorphic (Poly, n = 18, □) and macular (Mac, n = 10, ■) PKDL. iii Bar graphs showing the median (IQR) of anti-leishmanial IgM at disease presentation in patients with polymorphic (Poly, n = 24, □) and macular (Mac, n = 22, ■) PKDL, and after 3 weeks treatment (t/t) with LAmB (Poly, n = 21, □) and (Mac, n = 19, ■). **D: Effect of treatment upon antileishmanial IgG1 in PKDL** i Scatter plots indicating the median (IQR) of IgG1 in patients with polymorphic PKDL (n = 38, ■), macular PKDL (n = 26, □) and healthy controls HC (n = 15, ●) ii Bar graphs showing the median (IQR) of anti-leishmanial IgG1 before (pre) and after treatment (t/t) with 12 weeks Miltefosine in patients with polymorphic (Poly, n = 18, □) and macular (Mac, n = 10, ■) PKDL. iii Bar graphs showing the median (IQR) of anti-leishmanial IgG1 at disease presentation (pre) in patients with polymorphic (Poly, n = 24, □) and macular (Mac, n = 22, ■) PKDL, and after 3 weeks treatment (t/t) with LAmB (Poly, n = 21, □) and (Mac, n = 19, ■). **E: Effect of treatment upon levels of antileishmanial IgG2 in PKDL**. i Scatter plots indicating the median (IQR) of IgG2 in patients with polymorphic PKDL (n = 38, ■), macular PKDL (n = 26, □) and healthy controls HC (n = 15, ●). ii Bar graphs showing the median (IQR) of anti-leishmanial IgG2 before (pre) and after treatment (t/t) with 12 weeks Miltefosine in patients with polymorphic (Poly, n = 18, □) and macular (Mac, n = 10, ■) PKDL. iii Bar graphs showing the median (IQR) of anti-leishmanial IgG2 at disease presentation (pre) in patients with polymorphic (Poly, n = 24, □) and macular (Mac, n = 22, ■) PKDL, and after 3 weeks treatment (t/t) with LAmB (Poly, n = 21, □) and (Mac, n = 19, ■). **F: Effect of treatment upon levels of antileishmanial IgG3 in PKDL** i Scatter plots indicating the median (IQR) of IgG3 in patients with polymorphic PKDL (n = 38, ■), macular PKDL (n = 26, □) and healthy controls HC (n = 15, ●). ii Bar graphs showing the median (IQR) of antileishmanial IgG3 before (pre) and after treatment (t/t) with 12 weeks Miltefosine in patients with polymorphic (Poly, n = 18, □) and macular (Mac, n = 10, ■) PKDL. iii Bar graphs showing the median (IQR) of antileishmanial IgG3 at disease presentation (pre) in patients with polymorphic (Poly, n = 24, □) and macular (Mac, n = 22, ■) PKDL, and after 3 weeks treatment (t/t) with LAmB (Poly, n = 21, □) and (Mac, n = 19, ■).

The spectrum of anti-leishmanial immunoglobulins included Ig, IgG (and its subclasses, IgG1, IgG2 and IgG3) along with IgM, and levels in healthy non endemic individuals represented the baseline, whose levels of Ig, IgG and IgM were 0.37[0.24–0.39], 0.11[0.09–0.18] and 0.20[0.14–0.30] respectively (**Fig 2A–2Ci**); the IgG1, IgG2 and IgG3 levels were 0.20[0.12–0.29], 0.24[0.18–0.33] and 0.17[0.11–0.21] respectively (**Fig 2D–2Fi**).

PKDL cases with polymorphic features had a higher proportion of anti-leishmanial Ig in polymorphic (n = 38, 0.65[0.42–1.18], p<0.001) than the macular forms (n = 26, 0.59[0.43–0.88], p<0.01, **Fig 2Ai**) in comparison to healthy controls. Following random allocation to receive Miltefosine or LAmB, polymorphic cases who received Miltefosine showed a significant 2.8 fold decrease in total Ig from 1.07[0.63–1.48] to 0.38[0.26–0.73], p<0.01, whereas in the macular form the decrease was marginal from 0.64[0.57–0.94] to 0.49[0.28–0.78] **(Fig 2Aii**); with LAmB irrespective of clinical forms were unchanged (**Fig 2Aiii**).

To delineate the major contributor, IgG and IgM levels were measured, wherein polymorphic and macular cases showed a significant 5.7 fold and 4.3 fold increase in IgG compared to healthy controls, being 0.63[0.42–1.01], p<0.001 and 0.48[0.33–0.82], p<0.001 respectively (**Fig 2Bi**). Miltefosine decreased IgG in both forms, being 1.00[0.82–1.13] to 0.55[0.31–0.71], p<0.01 and, 0.49 [0.39–1.12] to 0.27 [0.22–0.75] in polymorphic and macular cases respectively (**Fig 2Bii).** However, in cases who received LAmB, their IgG levels remained unchanged (**Fig 2Biii).**

With regard to anti-leishmanial IgM, it was significantly increased in polymorphic 0.41[0.24–0.59], p<0.01 and macular PKDL, 0.34[0.25–0.56], p<0.05, (**Fig 2Ci**). Akin to IgG, Miltefosine caused a 2.2 fold decrease in polymorphic, 0.41[0.20–0.51] vs. 0.18 [0.08–0.59] and 2.3 fold in macular cases, 0.41[0.25–0.78] vs. 0.18[0.12–0.20], p<0.05 (**Fig 2Cii**). However, with LAmB, irrespective of the clinical form, IgM remained unchanged, (**Fig 2Ciii).**

In view of the considerable increase in IgG, measurement of IgG subclasses namely IgG1, IgG2 and IgG3 was measured. In polymorphic and macular PKDL, there was a significant elevation of IgG1, being 0.54[0.33–0.75] p<0.001 and 0.44[0.35–0.77], p<0.001 respectively (**Fig 2Di**). Miltefosine significantly decreased IgG1 in polymorphic cases from 0.51[0.31–0.81] to 0.24[0.17–0.29], p<0.01 and in macular cases from 0.41[0.34–0.60] to 0.22[0.15–0.34], p<0.05 (**Fig 2Dii**). However, with LAmB, the levels remained unchanged (**Fig 2Diii**).

Similarly with regard to IgG2, the polymorphic and macular cases demonstrated a significant increase, being 0.54[0.34–0.67], p<0.001 and 0.45[0.31–0.59], p<0.001 (**Fig 2Ei**) respectively. With Miltefosine, IgG2 decreased significantly in the polymorphic cases from 0.57[0.37–0.65] to 0.29[0.21–0.42], p<0.05, but only marginally in the macular group 0.42[0.31–0.57] to 0.31[0.15–0.38], (**Fig 2Eii)**, while LAmB failed to impact on IgG2 (**Fig 2Eiii**).

With regard to IgG3, there was a significant elevation in polymorphic, 0.57[0.34–0.78], p<0.001 and macular forms, 0.65[0.40–1.06], p<0.001 (**Fig 2Fi**). Importantly, Miltefosine significantly curtailed IgG3 in polymorphic, 0.62[0.39–0.86] to 0.20[0.14–0.36], p<0.001 and macular cases, 0.70[0.51–1.13] to 0.34[0.23–0.43], p<0.05, (**Fig 2Fii**). This was not evident in cases treated with LAmB as IgG3 remained unchanged (**Fig 2Fiii**).

### Longitudinal monitoring of total Ig, IgG, IgM and IgG subclasses

**Fig 3:**
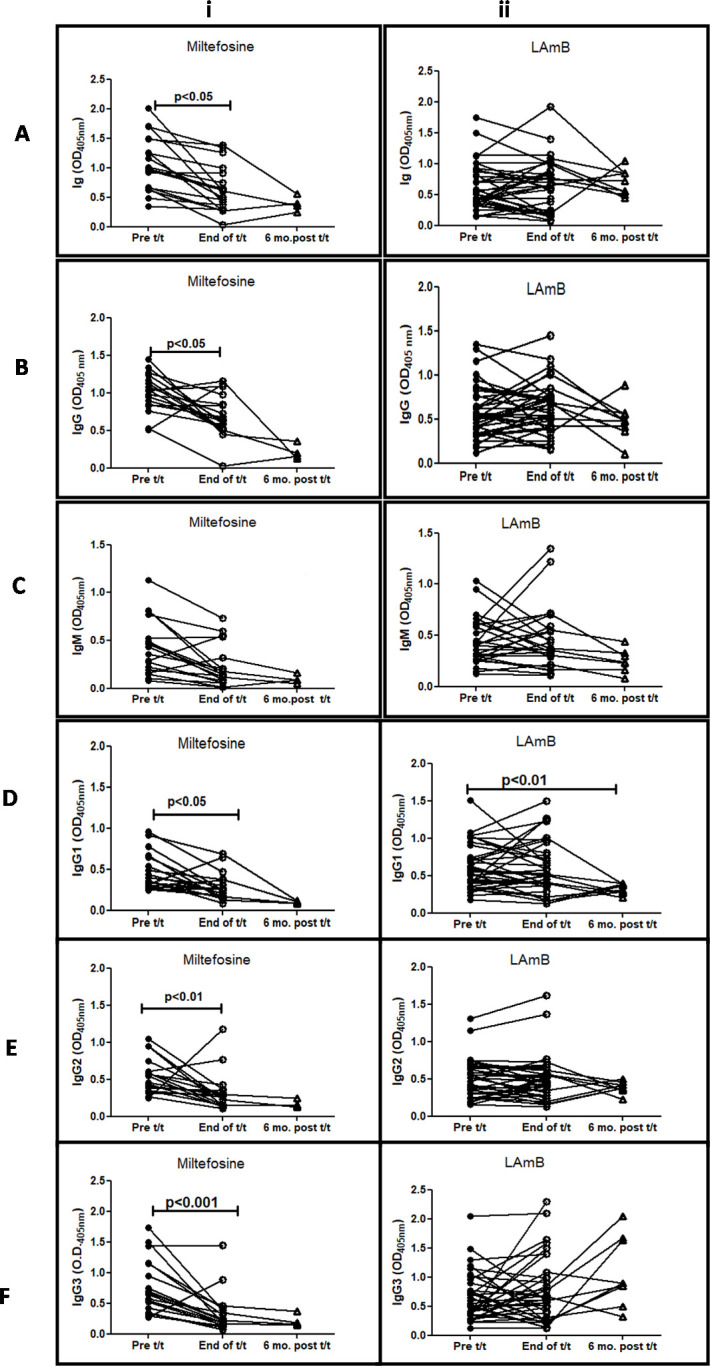
Longitudinal monitoring of anti-leishmanial Ig, IgG and IgM in patients with PKDL treated with Miltefosine or LAmB. **A: Serial monitoring of anti-leishmanial Ig.** i Before after plots indicating the plasma levels of total Ig at disease presentation (Pre, n = 18, ●), end of treatment with Miltefosine (End of t/t, n = 18, ○) and six months later (6 mo.post t/t, n = 4, Δ). ii Before after plots indicating the plasma levels of total Ig at disease presentation (pre, n = 36, ●), end of treatment with LAmB (End of t/t, n = 36, ○) and six months later (6 mo.post t/t, n = 9, Δ). **B**: **Serial monitoring of anti-leishmanial IgG**. i Before after plots showing the plasma levels of IgG at disease presentation (Pre, n = 18, ●), end of treatment with Miltefosine (End of t/t, n = 18, ○) and six months later (6 mo.post t/t, n = 4, Δ). ii Before after plots showing plasma levels of IgG at disease presentation (Pre, n = 36, ●), end of treatment with LAmB (End of t/t, n = 36, ○) and six months later (6 mo.post t/t, n = 9, Δ). **C**: **Serial monitoring of anti-leishmanial IgM**. i Before after plots showing the plasma levels of IgM at disease presentation (Pre, n = 18, ●), end of treatment with Miltefosine (End of t/t, n = 18, ○) and six months later (6 mo.post t/t, n = 4, Δ). ii Before after plots showing plasma levels of IgM at disease presentation (Pre, n = 36, ●), end of treatment with LAmB (End of t/t, n = 36, ○) and six months later (6 mo.post t/t, n = 9, Δ). **D: Serial monitoring of anti-leishmanial IgG1** i Before after plots indicating the plasma levels of IgG1 at disease presentation (Pre, n = 18, ●), end of treatment with Miltefosine (End of t/t, n = 18, ○) and six months later (6 mo.post t/t, n = 4, Δ). ii Before after plots indicating the plasma levels of IgG1 at disease presentation (Pre, n = 36, ●), end of treatment with LAmB (End of t/t, n = 36, ○) and six months later (6 mo.post t/t, n = 9, Δ). **E: Serial monitoring of anti-leishmanial IgG2**. i Before after plots indicating the plasma levels of IgG2 at disease presentation (Pre, n = 18, ●), end of treatment with Miltefosine (End of t/t, n = 18, ○) and six months later (6 mo.post t/t, n = 4, Δ). ii Before after plots indicating the plasma levels of IgG2 at disease presentation (Pre, n = 36, ●), end of treatment with LAmB (End of t/t, n = 36, ○) and six months later (6 mo.post t/t, n = 9, Δ). **F: Serial monitoring of anti-leishmanial IgG3.** i Before after plots indicating the plasma levels of IgG3 at disease presentation (Pre, n = 18, ●), end of treatment with Miltefosine (End of t/t, n = 18, ○) and six months later (6 mo.post t/t, n = 4, Δ). ii Before after plots indicating the plasma levels of IgG3 at disease presentation (Pre, n = 36, ●), end of treatment with LAmB (End of t/t, n = 36, ○) and six months later (6 mo.post t/t, n = 9, Δ).

Following treatment with Miltefosine (n = 18), serial monitoring was performed at three time points (i) presentation (ii) on completion of treatment, and (iii) six months later (wherever possible). On completion of 12 weeks treatment, the total Ig decreased significantly and six months later, there was an additional 1.5 fold decrease (**Fig 3Ai and Table A in S1 Table**). However, in 36 patients who received LAmB, Ig levels failed to decrease at the end of treatment and importantly, at the end of 6 months, remained high in the majority of cases (**Fig 3Aii and Table B in S1 Table**).

With regard to IgG, Miltefosine demonstrated a significant 1.6 fold decrease, which six months later decreased further by 3.7 fold (**Fig 3Bi and Table A in S1 Table**). whereas with LAmB, IgG levels remained unchanged even six months later (**Fig 3Bii and Table B in S1 Table**). Although Miltefosine caused a significant 3.0 fold decrement in IgM, which by the end of six months decreased a further 1.4 fold (**Fig 3Ci and Table A in S1 Table**), LAmB failed to alter IgM, even at the end of six months (**Fig 3Cii and Table B in S1 Table**).

Serial monitoring of IgG subclasses was done in patients treated with miltefosine (n = 18) or LAmB (n = 36)wherein IgG1 decreased by 1.5 fold with Miltefosine, and in four patients who were monitored six months later, it decreased further by 2.8 fold (**Fig 3Di and Table A in S2 Table**). With LAmB, at the end of 3 weeks treatment, the IgG1 levels remained unchanged, whereas at six months post-treatment (n = 9), a significant 1.8 fold decrease was evident (**Fig 3Dii and Table B in S2 Table**) and was attributed to 6/9 (66%) cases. However in 3/9 (33%) cases, there was an increase in IgG1 levels (**Fig 3Dii)**. Similarly, with regard to IgG2, Miltefosine caused a sustained decrease (**Fig 3Ei and Table A in S2 Table**), whereas LAmB failed to decrease IgG2 levels, and even six months later, decreased marginally (**Fig 3Eii and Table B in S2 Table**). With regard to IgG3, Miltefosine caused a significant 3.0 fold decrement, which six months later, decreased an additional 1.4 fold (**Fig 3Fi and Table A in S2 Table**). However, with LAmB there was no decrease in IgG3 at the end of 3 weeks treatment; importantly, six months later IgG3 increased in 8/9 cases (**Fig 3Fii and Table B in S2 Table**).

### Longitudinal monitoring of IL-10 and IL-6 in PKDL

**Fig 4:**
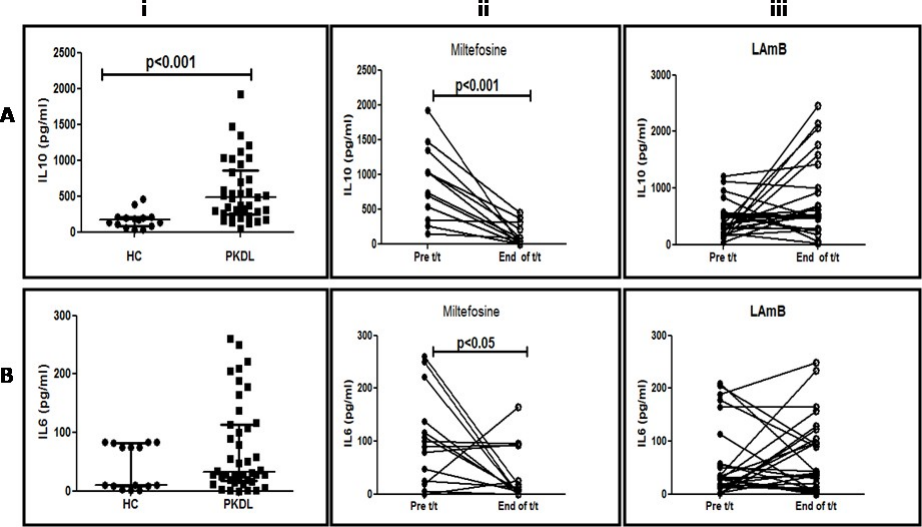
Levels of circulating cytokines following treatment with Miltefosine or LAmB. **A: Levels of IL-10.** i Scatter plots showing the median (IQR) levels of IL-10 in healthy controls (HC, n = 15, ●) and patients with PKDL (n = 38, ■). ii Before after plots of plasma IL10 at disease presentation (Pre t/t, n = 12, ●) and end of treatment with Miltefosine (End of t/t, n = 12, ○). iii Before after plots of plasma IL-10 at disease presentation (Pre t/t, n = 26, ●), end of treatment with LAmB (End of t/t, n = 26, ○). **B: Levels of IL-6**. i Scatter plots showing the median (IQR) levels of IL-6 in healthy controls (HC, n = 15, ●) and patients with PKDL (n = 42, ■). ii Before after plots indicating the plasma levels of total IL-6 at disease presentation (Pre t/t, n = 14, ●), end of treatment with Miltefosine (End of t/t, n = 14, ○). iii Before after plots indicating the plasma levels of total IL-6 at disease presentation (Pre t/t, n = 28, ●), end of treatment with LAmB (End of t/t, n = 28, ○).

Cytokines can mediate immunoglobulin class switching with IL-10 mediating the switch to IgG1 and IgG3, whereas IFN-γ with IL-6 can drive production of IgG2 [18]. Accordingly, as IgG1, IgG2 and IgG3 were increased (**Fig 2D–2F**), levels of IL-10 were measured in patients treated with Miltefosine (n = 12) or LAmB (n = 26). At disease presentation, the IL-10 levels were significantly elevated by 2.7 fold *vis-a-vis* healthy controls, being 481.5[251.7–862.4] vs. 174.3[80.0–205.0] pg/ml, p<0.001 (**Fig 4Ai).** In 12 serially monitored cases who received Miltefosine, there was an 11 fold decrease from 875.0[389.8–1271.0] to 80.0[0.0–283.6] pg/ml, p<0.001 (**Fig 4Aii).** However, with LAmB, the levels of IL-10 increased from 345.0[211.0–538.0] to 524.0[279.0–1104.0] pg/ml, as their levels either increased (n = 14, 54%), remained unaltered (n = 4, 15%) or decreased (n = 8, 31%, **Fig 4Aiii)**.

With regard to IL-6, there was a 3.2 fold elevation in patients with PKDL vs. healthy controls 32.0[18.1–113.8] vs. 10.0[8.9–82] pg/ml (**Fig 4Bi)**; with Miltefosine (n = 14), there was a significant 9.4 fold decrement from 94.5[23.1–158.7] to 10.4[0.0–92.5] pg/ml, p<0.05, (**Fig 4Bii),** whereas with LAmB (n = 28), the IL-6 levels remained unchanged being 28.1[15.3–57.3] and 38.6[8.0–103.2] pg/ml. In fact, their levels increased in (15/28, 53%) cases, remained unchanged (2/28, 8%) or decreased (11/28, 39%, **Fig 4Biii).**

**Fig 5:**
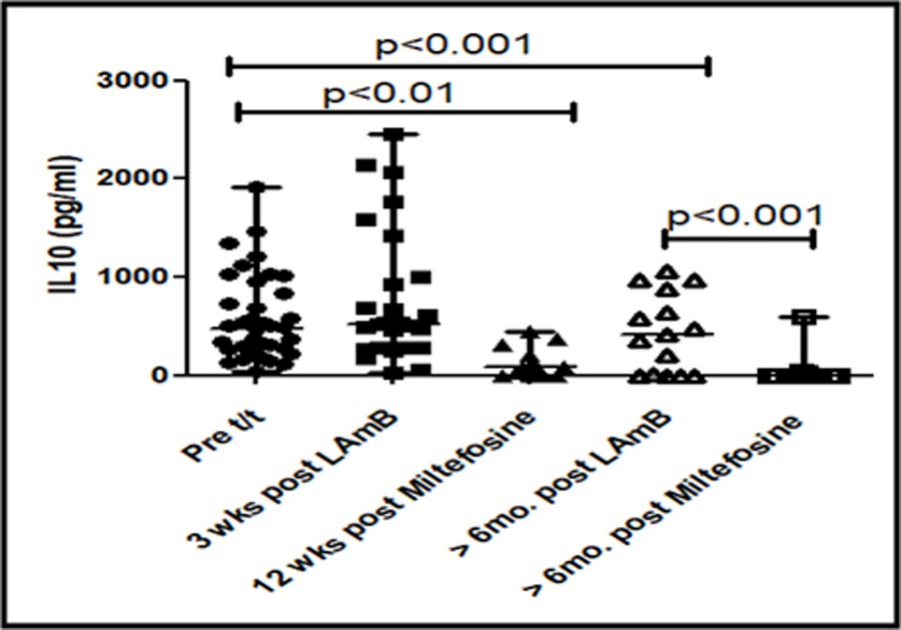
Kinetics of IL-10 in PKDL. Scatter plots indicating the median (IQR) of IL-10 in patients with PKDL at disease presentation (Pre, n = 38, ●, 1), end of t/t with LAmB (3 wks post LAmBn = 26, ■) or Miltefosine (12 wks post Miltefosine, n = 12,▲), six months post t/t with LAmB (>6 mo. post LAmB, n = 15, Δ) or Miltefosine (>6 mo. post Miltefoseine, n = 16, □).

**Fig 6:**
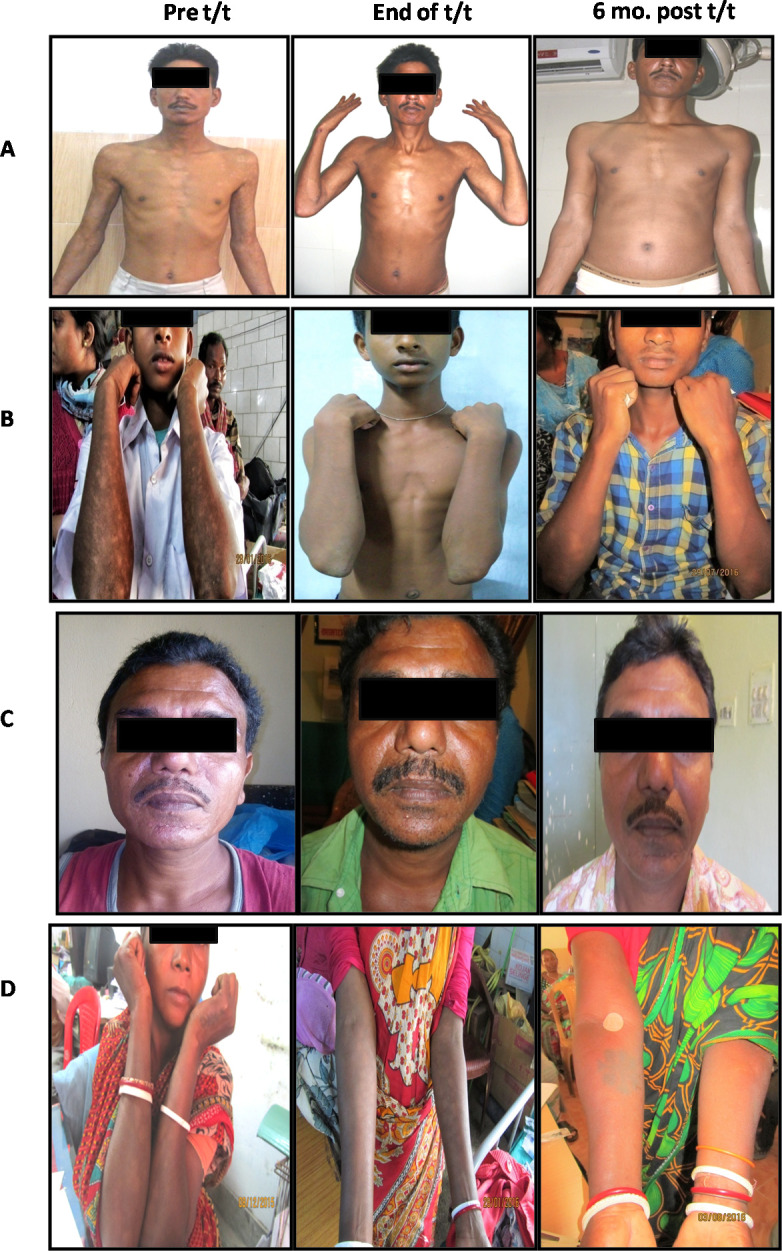
Status of dermal lesions in PKDL cases following treatment with Miltefosine or LAmB. **A, B:** Representative profiles of dermal lesions of a patient with polymorphic (**A**) or macular (**B**) PKDL at disease presentation (Pre t/t), following completion of 12 weeks treatment with Miltefosine (End of t/t) and six months later (6 mo. post t/t). **C, D**: Representative profiles of dermal lesions of a patient with polymorphic (**C**) or macular (**D**) PKDL at disease presentation (Pre t/t), following completion of 3 weeks treatment with LAmB (End of t/t) and six months later (6 mo.post t/t).

Additionally, the efficacy of IL-10 was confirmed by monitoring levels in randomly selected PKDL cases (n = 38), immediately after treatment with LAmB (n = 26) or Miltefosine (n = 12), and at any time point six months later. In the miltefosine treated group, at time points greater than 6 months t (n = 16), IL-10 was non-detectable in the majority of cases **(Fig 5),** and correlated with total disappearance of dermal lesions **(Fig 6A and 6B)** and absence of parasite load (**Fig 1**). However, the levels of IL-10 in patients who received LAmB when measured at or after six months (n = 15), did not demonstrate a reduction, being 410[0–876] pg/ml (**Fig 5),** and importantly, correlated with clinical features as their dermal lesions persisted (**Fig 6C and 6D),** and corroborated with a persisting parasite burden (**Fig 1**).

## Discussion

Disease progression in human VL has been consistently associated with an abundant production of Th1 (IFN-γ and TNF-α) and Th2 (TGF-β and IL-4) cytokines, with the latter coupled with IL-10 taking the upper hand [19,20]. Similarly in PKDL, a raised mRNA expression of both pro- and anti-inflammatory cytokines has been demonstrated at lesional sites [21–23] along with a raised mRNA expression of counter-regulatory cytokines, TGF-β and IL-10 [21,22,24–26], that collectively supported a pro-parasitic immunosuppressive milieu [27–29].

Although studies have proposed the ability of Th1 and Th2 cells to support B cell responses [30], the latter is generally accepted to be more adept in this respect, as validated by their cytokines being key contributors towards B cell proliferation and differentiation [31,32]. Accordingly, in Leishmaniasis, the Th2 predominant microenvironment stimulates B cell activation leading to secretion of IgG and enhanced isotype switching [17,33]. In VL, this paved the way for development of serological assays, with the rk39 ‘rapid diagnostic test’ revolutionizing diagnosis [34]. However, analogous tests for PKDL till date remain limited.

Ideally, monitoring the chemotherapeutic effectiveness of PKDL requires direct detection of parasite or its DNA, the latter being the first choice. However, there are logistic limitations, as once the lesions decline patients are reluctant to provide a repeat skin biopsy. Furthermore, although PCR is a clear winner [35,36], the associated expenses, availability of sophisticated facilities and trained personnel precludes its applicability in resource limited settings. An excellent alternative would be detection of parasite antigens and has been implemented in VL using urine-based assays. However, its moderate sensitivity [15 and references therein] necessitates that efforts be aimed at developing a non invasive ‘test of cure’, especially for PKDL.

Studies have alluded to the presence of a high proportion of anti-leishmanial antibodies in PKDL [33,37], but their potential in monitoring their treatment efficacy has not been investigated, and was the focus of this study. Anti-leishmanial antibody levels of total Ig, IgG, IgM IgG1, IgG2 and IgG3 were monitored in polymorphic and macular PKDL, and their raised levels (**Fig 2**) corroborated with previous studies [33,38,39]. Miltefosine impacted substantially upon all antibody subclasses especially in the polymorphic form, and accounted for the decrease in IgG (**Fig 2Aii**) as also IgG1, IgG2 and IgG3 (**Fig 2D–2F**). This was in agreement with the parasite load wherein Miltefosine caused a dramatic decline in parasite load, more so in the polymorphic form [13,14]. Miltefosine mediates its leishmanicidal activity directly via parasite apoptosis [40] and indirectly by its immunomodulatory ability to skew macrophages towards a M2 phenotype, accompanied by a decrease in circulating levels of IL-10, TGF-β and IL-4 [24] which in this study translated into a decrease in the levels of Immunoglobulins (**Fig 3**). However, it remains an open ended yet pertinent question as to why the macular type *vis-a-vis* the polymorphic form failed to demonstrate a similar decrease in antibody levels, notable exceptions being IgG1 and IgG3 (**Fig 2**).

IL-10 is a key cytokine that drives *Leishmania* infection [41] and based on its propensity to induce naive slgD^+^ B cells to secrete IgG1 and IgG3 [42], is responsible for the increased levels of IgG1 and IgG3 in PKDL (**Fig 1**). As Miltefosine is known to decrease IL-10 [24], it translated into lowering of levels of IgG1 and IgG3 (**Figs 2–4**). A similar scenario was reported in serially monitored patients with CL [43] and American VL [44], endorsing its applicability in monitoring chemotherapeutic responses in PKDL (**Fig 3**). This study also corroborated the chemotherapeutic superiority of Miltefosine *vis-à-vis* LAmB in PKDL [13], as antibody levels failed to decrease with LAmB (**Fig 3**). However, the study by Moulik et al., (2018) [13], had a limited number of samples especially at the time point of 6 months post treatment, owing to an inability to collect skin biopsies following completion of treatment. It is anticipated that a non-invasive approach as applied in this study can circumvent this problem. It can be justifiably argued that following three weeks of LAmB, antibody levels are unlikely to decrease, and measurement at a later time point would be more relevant. Accordingly, antibody levels in patients treated with LAmB were measured at 6 months post-treatment in 9 cases, wherein the IgG3 levels increased in 8/9 cases (**Fig 3**), Therefore, it is imperative such cases of treatment failure be identified, as they may require additional or alternative treatment, or else will become mobile disease reservoirs, and derail the progress of the ongoing VL elimination programme.

The superiority of Miltefosine over LAmB in PKDL has been reported [45], but the underlying reasons remain to be elucidated. As monocytes/macrophages at lesional sites are critical for delivering LAmB, the degree of infiltration could impact on its lesional concentration as validated in a murine model of CL [46]. Therefore, it may be suggested that in macular cases having a lower proportion of macrophage infiltration [47], translated into a lowered accumulation of LAmB, and contributed towards its reduced therapeutic response.

Levels of plasma IL-10 are raised in human VL [48,49] as also PKDL [25,26,28], and declined following treatment. Additionally, in PKDL, a raised mRNA/protein expression of IL-10 has been demonstrated in skin lesions [26]. Importantly, the significant decline in plasma IL-10 after treatment with Miltefosine indicated its strong association with parasite clearance and resolution of dermal lesions (**Figs 4 and 6**). Furthermore, the decrease in antibody levels was most prominent with IgG1 and IgG3, whose switching is mediated by IL-10 [42]. Additionally, in PKDL cases that received LAmB, the unchanged or increased levels of IL-10 (**Fig 3A**) coincided with their unchanged or increased levels of IgG3 (**Fig 3Fii**). Studies have reported a positive correlation between IL-10 and parasite load in human VL [50] and lesional tissue of PKDL patients [26]; accordingly, this study endorsed the translational potential of IL-10 and/or IgG3 as biomarkers for monitoring PKDL.

In patients with VL, increased levels of circulating IFNγ regularly accompany progressive infections as also cure, and was reflected in raised IgG2 and IgG4 at disease presentation and cure [17,25,39]. A similar scenario was reported in Disseminated Leishmaniasis wherein high levels of IFNγ were accompanied by an increased IgG2, and importantly, was an effective predictor of disease [51]. At disease presentation, the raised IFNγ reported in human VL [21,52,53], CL [54], and PKDL [21,41] indicates a mixed Th1/Th2 milieu, the latter being predominant [20–22]). Accordingly, as IFNγ drives the levels of IL-6, it accounted for the increase in IL-6 (**Fig 3B**) along with IgG2 which is dependent on IFNγ and IL-6 [18]. With Miltefosine, the resultant parasite elimination led to a decrease in IFNγ [55], and a concomitant decrease in IgG2 (**Figs 2E and 4B**). Moreover, the lack of efficacy observed with LAmB reflected in the unchanged or even raised levels of IL-6 and IgG2 (**Figs 4B and 3Eii**).

This study was an extension of a randomized sampling mode study where suspected cases of PKDL reported at a medical camp, and after confirmation of diagnosis were randomly allocated Miltefosine or LAmB depending on drug availability [13]. In 2010, the prevalence of PKDL was reported to range from 4.4 to 7.8 per 10,000 endemic population, and later declined to 1.1 per 10,000 population in a 2017 study [56 and references therein]. Accordingly, taking a real world, pragmatic approach, sample size calculation was not attempted. Another drawback of this study was irrespective of treatment received, there was a high attrition rate, especially at six months post-treatment (**Fig 1**), and therefore, no statistical analysis in terms of paired data was attempted at the 6 months time point. Although detection of LD bodies was independently performed anonymously by a pathologist, other laboratory diagnostic tests were not carried out in a blinded fashion, and therefore a potential for bias existed.

The overall management, chemotherapy and control of transmission of Leishmaniasis largely depend on the availability of robust diagnostic tools that can provide early and unequivocal results. The success of the ongoing kala-azar elimination programme requires addressing the last mile challenges that include availability of tools for monitoring therapy and epidemiological surveillance to allow for prompt detection of potential outbreaks. It would therefore be prudent to consider a non-invasive approach such as measurement of circulating IgG3 and IL-10 to identify patients with PKDL who fail to respond to chemotherapy.

## Supporting information

S1 Table Impact of Miltefosine (A) or LAmB (B) upon levels of antileishmanial Ig, IgG and IgM in PKDL.
(DOC)

S2 Table Monitoring of anti-leishmanial IgG subclasses (IgG1, IgG2 and IgG3) in patients with PKDL following treatment with (A) Miltefosine or (B) LAmB.
(DOC)
