# Supplementary material for: IgG3 and IL10 are effective biomarkers for monitoring therapeutic effectiveness in Post Kala-Azar Dermal Leishmaniasis
Source: PLoS Negl Trop Dis. 2021 Nov 10;15(11):e0009906. doi: 10.1371/journal.pntd.0009906 (PMC8580238; doi:10.1371/journal.pntd.0009906)
Supplement: S1 Table — Impact of Miltefosine (A) or LAmB (B) upon levels of antileishmanial Ig, IgG and IgM in PKDL. (DOC) [file pntd.0009906.s001.doc]

**S1 Table: Impact of anti-leishmanial therapy upon levels of antileishmanial Ig, IgG and IgM in PKDL**

**A: Impact of Miltefosine**

| **Anti-leishmanial**  **Immunoglobulin Levels** | ***Presentation** | ***End of treatment**  **(Miltefosine)** | ***6 months later** |
| --- | --- | --- | --- |
| Ig | 0.99 [0.65-1.48] | 0.55 [0.29-0.73]^a^ | 0.37 [0.27-0.51] |
| IgG | 1.03 [0.83-1.15] | 0.63 [0.52-0.88]^a^ | 0.17 [0.13-0.31] |
| IgM | 0.39 [0.18-0.58] | 0.13[0.07-0.37] | 0.09 [0.06-0.11] |

**B: Impact of LAmB**

| **Anti-leishmanial Immunoglobulin Levels** | ***Presentation** | ***End of treatment**  **(LAmB)** | ***6 months later** |
| --- | --- | --- | --- |
| Ig | 0.52 [0.37-0.89] | 0.64 [0.20-0.89] | 0.72 [0.51-0.84] |
| IgG | 0.53 [0.35-0.76] | 0.63 [0.41-0.75] | 0.47 [0.38-0.55] |
| IgM | 0.40 [0.26-0.60] | 0.36 [0.26-0.56] | 0.24 [0.16-0.32] |

*Values are expressed as median (IQR) of absorbances measured at 405 nm as described in Materials & methods; ^a^p<0.05: significantly different from presentation.
