# Supplementary material for: IgG3 and IL10 are effective biomarkers for monitoring therapeutic effectiveness in Post Kala-Azar Dermal Leishmaniasis
Source: PLoS Negl Trop Dis. 2021 Nov 10;15(11):e0009906. doi: 10.1371/journal.pntd.0009906 (PMC8580238; doi:10.1371/journal.pntd.0009906)
Supplement: S2 Table — Monitoring of anti-leishmanial IgG subclasses (IgG1, IgG2 and IgG3) in patients with PKDL following treatment with (A) Miltefosine or (B) LAmB. (DOC) [file pntd.0009906.s002.doc]

**S2 Table: Monitoring of anti-leishmanial IgG subclasses (IgG1, IgG2 and IgG3) in patients with PKDL following treatment.**

(A) Miltefosine

| **Antileishmanial IgG subclass Levels** | ***Presentation** | ***End of treatment**  (Miltefosine) | ***6 months later** |
| --- | --- | --- | --- |
| IgG1 | 0.37 [0.29-0.64] | 0.25 [0.17-0.37]^a^ | 0.09 [0.08-0.11] |
| IgG2 | 0.50 [0.34-0.64] | 0.28 [0.15-0.35]^b^ | 0.15 [0.13-0.22] |
| IgG3 | 0.66 [0.39-1.14] | 0.22 [0.16-0.39]^c^ | 0.16 [0.15-0.32] |

**B: LAmB**

| **Antileishmanial IgG subclass levels** | ***Presentation** | ***End of treatment** | ***6 months later** |
| --- | --- | --- | --- |
| IgG1 | 0.54 [0.36-0.74] | 0.51 [0.35-0.80] | 0.30 [0.27-0.38]^b^ |
| IgG2 | 0.48 [0.30-0.65] | 0.44 [0.29-0.60] | 0.38 [0.35-0.44] |
| IgG3 | 0.51 [0.35-0.86] | 0.61 [0.27-0.99] | 0.89 [0.67-1.65] |

*Values are expressed as median (IQR) of absorbances measured at 405 nm as described in Materials & methods; ^a^p<0.05, ^b^p<0.01, ^c^p<0.001: significantly different from presentation.
